# A new species of *Melithaea* (Anthozoa, Octocorallia, Melithaeidae) from the Oman Sea, off Oman

**DOI:** 10.3897/zookeys.623.10045

**Published:** 2016-10-11

**Authors:** Kaveh Samimi-Namin, Leen P. van Ofwegen, Catherine S. McFadden

**Affiliations:** 1 Naturalis Biodiversity Center, PO Box 9517, 2300 RA Leiden, the Netherlands; 2 Department of Biology, Harvey Mudd College, 1250 N. Dartmouth Ave., Claremont, CA 91711 USA

**Keywords:** Persian Gulf, octocorals, Indian Ocean, Middle East, northwest Indian Ocean

## Abstract

A new species, *Melithaea davidi* **sp. n.**, is described from the eastern coast of Oman, Oman Sea, in the northwestern Indian Ocean, where it differs from its congeners in lacking capstans and having predominantly spindles in the coenenchyme. A molecular phylogenetic analysis of mtMutS and 28S rDNA genes suggests that it is genetically distinct from similar species in the Red Sea. Furthermore, a species previously reported as *Acabaria* sp. from the Arabian Sea is now identified as *Melithaea mabahissi* (Hickson, 1940).

## Introduction

Melithaeidae, one of the 49 presently recognized families of octocorals (Ofwegen and McFadden 2010; McFadden and Ofwegen 2012, 2013; Breedy et al. 2012), has only two valid genera (Reijnen et al. 2014) and numerous species distributed worldwide. They are sessile, benthic, and colonial organisms that require a hard substrate for settlement and anchorage. The melithaeid species are widespread in marine environments, from shallow to deep sea, but are most abundant in warm and tropical waters (Fabricius and Alderslade 2001). Their geographical distribution ranges from East Africa and the Red Sea (Grasshoff 2000), Indian Ocean (Thomson 1916; Ofwegen 1987, 1989; Williams 1992), Indo-West Pacific (Ofwegen 1987; Grasshoff 1999; Ofwegen et al. 2000; Hoeksema and van Ofwegen 2004, 2008; Matsumoto and Ofwegen 2015) to Hawaii (Bayer 1956). In spite of this vast distribution range, only a few species of melithaeids have been reported from the northwestern Indian Ocean (the coasts of the Arabian Sea, the Red Sea, the Gulf of Aden, the Oman Sea, and the Persian Gulf). These include *Clathraria omanensis* off Oman in the Oman Sea, *Acabaria* spec. indet. 2 from West India in the Arabian Sea (Ofwegen 1987), *Acabaria* spec. indet. 1 from Kenya and Somalia, and *Acabaria mabahissi* from Somalia (Hickson 1940). Although no melithaeid species have so far been recorded and sampled from the shallow coastal waters of the Persian Gulf (Samimi-Namin and van Ofwegen 2009, 2012), several colonies have been distinguished in video footage and photographs taken from mesophotic depths (> 40 m) of the Strait of Hormuz (pers. obs.).

Melithaeidae is an uncommon family in the north-western Indian Ocean, and its maximum depth extends beyond that of conventional diving activities. This might explain the scarce records and rarity of these species within this region where they have not been documented in major coral studies (see Sheppard and Salm 1988; Sheppard and Sheppard 1999; Sheppard et al. 2000; Claereboudt 2006; Samimi-Namin and van Ofwegen 2016a, b). In contrast, this family is relatively common in the shallow warm waters of the central Indo-Pacific and the Coral Triangle (Fabricius and Alderslade 2001; Hoeksema and van Ofwegen 2004, 2008).

It is known that the Arabian Sea and Oman Sea have a complex hydrography, mainly caused by seasonal monsoons. The summer southwest monsoon generates one of the five largest upwelling areas of the world (Bakun et al. 1998), whereas the winter northeast monsoon reverses the circulation pattern and increases the biological production of the whole northern Indian Ocean (Burkill 1999, Wilson 2000). In spite of these hydrographical and geological complexities which might induce high endemicity and diversity, there are still many species and habitats yet to be discovered and documented from shallow and deep waters of the north-western Indian Ocean. Traditionally six genera have been recognized in Melithaeidae based on the sclerite morphology of their coenenchyme: *Acabaria* Gray, 1859 and *Asperaxis* Alderslade, 2006, both with a variety of spindles and occasionally a few thorn-clubs; *Clathraria* Gray, 1859, with a complete layer of capstans, and double heads; *Melithaea* Linnaeus, 1758, with predominantly asymmetrical double-discs, a few leaf-clubs and thorn-clubs; *Mopsella* Gray, 1857, with leaf-clubs and thorn-clubs at the surface of the coenenchyme; and *Wrightella* Gray, 1870, with foliate spheroids (Hickson 1937; Ofwegen 1987, Grasshoff 2000). In the literature, numerous species have been described as being intermediate between these genera, with considerable variation and overlap in their sclerite morphology. Several researchers have pointed this out and have suggested a revised classification system for this family (Grasshoff 1999, 2000; Fabricius and Alderslade 2001; Reijnen et al. 2014). Although the latest study by Reijnen et al. (2014) considers all the genera in the classification proposed by Hickson (1937) as variations across geographical regions and suggests the merger of all these genera into one, i.e. *Melithaea*, the taxonomic situation of this group of octocorals has not been resolved satisfactorily and the family has to be formally revised. Based on the previous taxonomic system, an undescribed species discovered in the Oman Sea falls into a group with the coenenchyme predominated by spindles and occasionally thorn-clubs (*Acabaria*/*Asperaxis*). However, to avoid further confusion we follow Reijnen et al. (2014) regarding generic classification, considering two valid genera in the Melithaeidae: *Melithaea*, and *Asperaxis*, with the latter genus only reported from Australia.

In this paper, we describe a new species of the genus *Melithaea* from approximately 80 m depth, off the coast of Oman.

### Abbreviations

NBC
Naturalis Biodiversity Center, Leiden, The Netherlands; previously National Museum of Natural History
(NNM); formerly Rijksmuseum van Natuurlijke Historie (RMNH)

RMNH Rijksmuseum van Natuurlijke Historie, Leiden, currently NBC

UNESCO-IOC United Nations Educational, Scientific and Cultural Organization- Intergovernmental Oceanographic Commission

ZMTAU
Zoological Museum, Tel Aviv University.

## Material and methods

**Figure 1.**
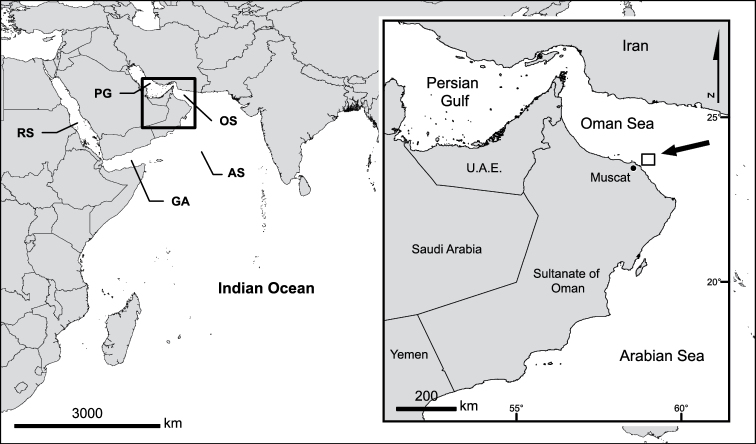
Type locality of *Melithaea davidi* sp. n. in the Oman Sea, off Muscat governorate. RS = Red Sea, PG = Persian Gulf, GA = Gulf of Aden, OS = Oman Sea, AS = Arabian Sea.

*In situ* observations and material collection was conducted in 2013, during a deep water dive in the Oman Sea (Fig. 1). *In situ* photographs were taken using a small compact underwater camera and the depth recorded using a dive computer. In total three colonies were collected and were preserved in ethanol. In order to identify the material, sclerites were obtained by dissolving the tissues in 10% sodium hypochlorite, followed by rinsing in fresh water. For scanning electron microscopy (SEM), the sclerites were carefully rinsed with double-distilled water, dried at room temperature, mounted on a stub with double-sided carbon tape, then coated with gold-palladium (AuPd), and examined using a Jeol 6480LV SEM operated at 10 kV.

All specimens are deposited at Naturalis Biodiversity Center, Leiden, the Netherlands (formerly Rijksmuseum van Natuurlijke Historie, Leiden, the Netherlands).

### Molecular and phylogenetic analyses

DNA was extracted from specimen RMNH Coel. 42122 using the Qiagen DNEasy Blood & Tissue Kit. Published primers and protocols (Reijnen et al. 2014) were used to obtain DNA sequences for fragments of the mitochondrial mtMutS (486 nt) and nuclear 28S rDNA (670 nt) genes. The two genes were aligned with the concatenated mtMutS plus 28S sequences of Reijnen et al. (2014), and the dataset was then re-analysed using both maximum likelihood and Bayesian approaches. Maximum likelihood trees were constructed using PhyML (Guindon and Gascuel 2003) with the GTR+I+Γ model of evolution and 100 bootstrap replicates. Bayesian analyses were run using MrBayes v. 3.2.1 (Ronquist et al. 2012) with that same model of evolution applied. Analyses were run for 2 million generations (until standard deviation of split partitions < 0.01) with a burn-in of 25% and default Metropolis coupling parameters. MEGA v.5 (Tamura et al. 2011) was used to calculate pairwise measures of genetic distance (Kimura 2-parameter) among sequences.

### Molecular and phylogenetic results

**Figure 2.**
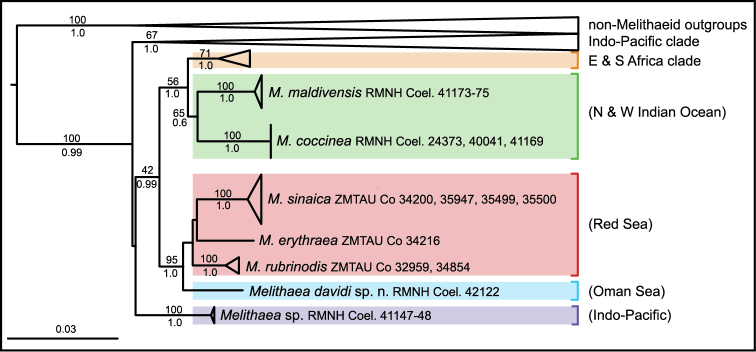
Maximum likelihood phylogeny of family Melithaeidae based on concatenated nucleotide sequences for mtMutS (486 nt) and 28S rDNA (670 nt) (sequence data for all but RMNH Coel. 42122 are from Reijnen et al. 2014). Well-supported clades of species from the Indo-Pacific, east and south Africa, and outgroup taxa (*Annella*, *Chironephthya*, *Euplexaura*, *Siphonogorgia*, *Solenocaulon*) have been collapsed to improve readability. Numbers above branches are maximum likelihood bootstrap percentages; numbers below branches are Bayesian posterior probabilities. Generic assignments follow the recommendations of Reijnen et al. (2014).

Maximum likelihood and Bayesian analyses of mtMutS plus 28S rDNA recovered identical tree topologies (Fig. 2) and strong to moderate support for the same geographically structured clades found by Reijnen et al. (2014) in their analysis of a four-gene dataset. Specimen RMNH Coel. 42122 fell within a well-supported clade of species from the Red Sea including *Melithaea erythraea* (Ehrenberg, 1834), *Melithaea sinaica* (Grasshoff, 2000), and *Melithaea rubrinodis* (Gray, 1859). It was well differentiated genetically from all three Red Sea species, differing from them by average genetic distance values (Kimura 2-parameter) ranging from 0.6–2.5% at mtMutS and 2.8–3.45% at 28S, values that are greater than those typically observed among conspecific octocorals (McFadden et al. 2014).

## Morphological descriptions and systematic account

### Class Anthozoa Ehrenberg, 1831 Subclass Octocorallia Haeckel, 1866 Order Alcyonacea Lamouroux, 1812 Family Melithaeidae Gray, 1870 Subfamily Melithaeinae Alderslade, 2006

#### Genus Melithaea

Taxon classificationAnimaliaAlcyonaceaMelithaeidae

Milne Edwards, 1857

##### Diagnosis.

Colonies with segmented axis, and swollen nodes and straight internodes containing cigar-shaped sclerites. Densely branched in one or more planes, forming large fans or forming bushes. Sclerites of coenenchymal surface are spindles, thorn-clubs, double discs, leaf clubs, and foliate spheroids. Polyps monomorphic, small and retractile. Calyces can be low or tall. Polyps contain spindle-like and club-like forms arranged as collaret and points, with dragon wing sclerites (flattened, more or less twisted, boomerang-shaped platelets commonly with the convex edge serrated near the wider end; present in the proximal part of tentacles/see Grasshoff 1999, 2000) in the tentacles. The colonies can be yellow, orange, red, dark purple, pink, and white. Axes are usually coloured, often red. Azooxanthellate.

#### Melithaea davidi sp. n.

Taxon classificationAnimaliaAlcyonaceaMelithaeidae

http://zoobank.org/62E50344-EFEF-4F94-8007-346D0AE2A9EF

**Figure 3.**
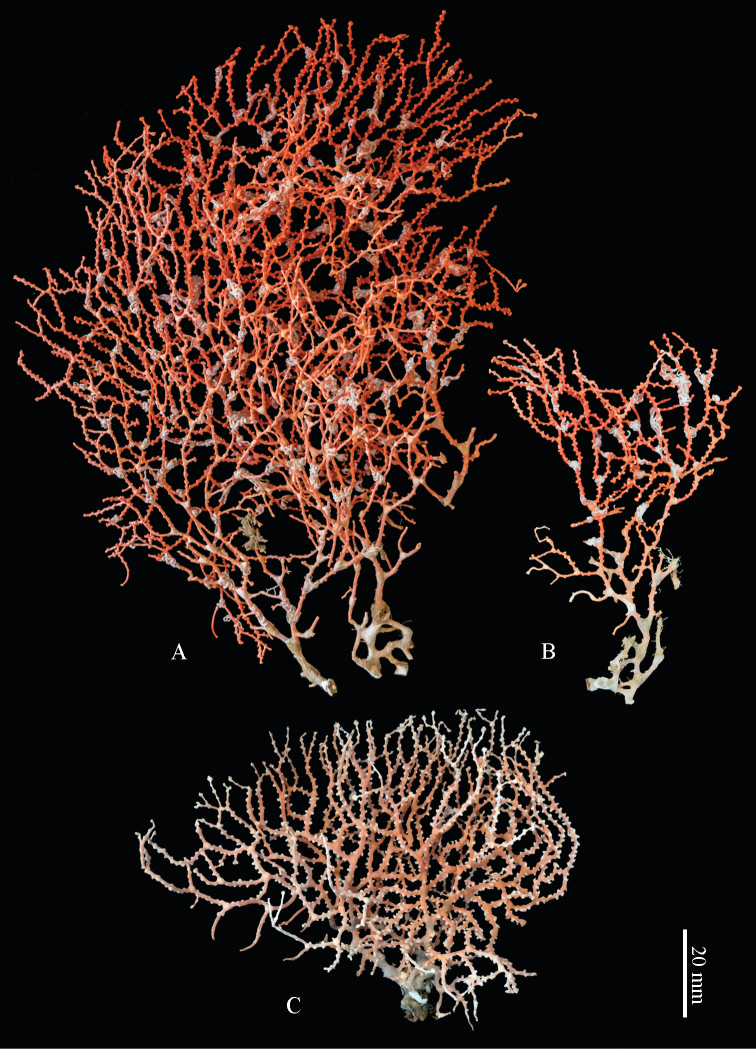
Colonies of *Melithaea davidi* sp. n.; **A** holotype, RMNH Coel. 42122 **B** paratype, RMNH Coel. 42123 **C** paratype, RMNH Coel. 42124.

**Figure 4.**
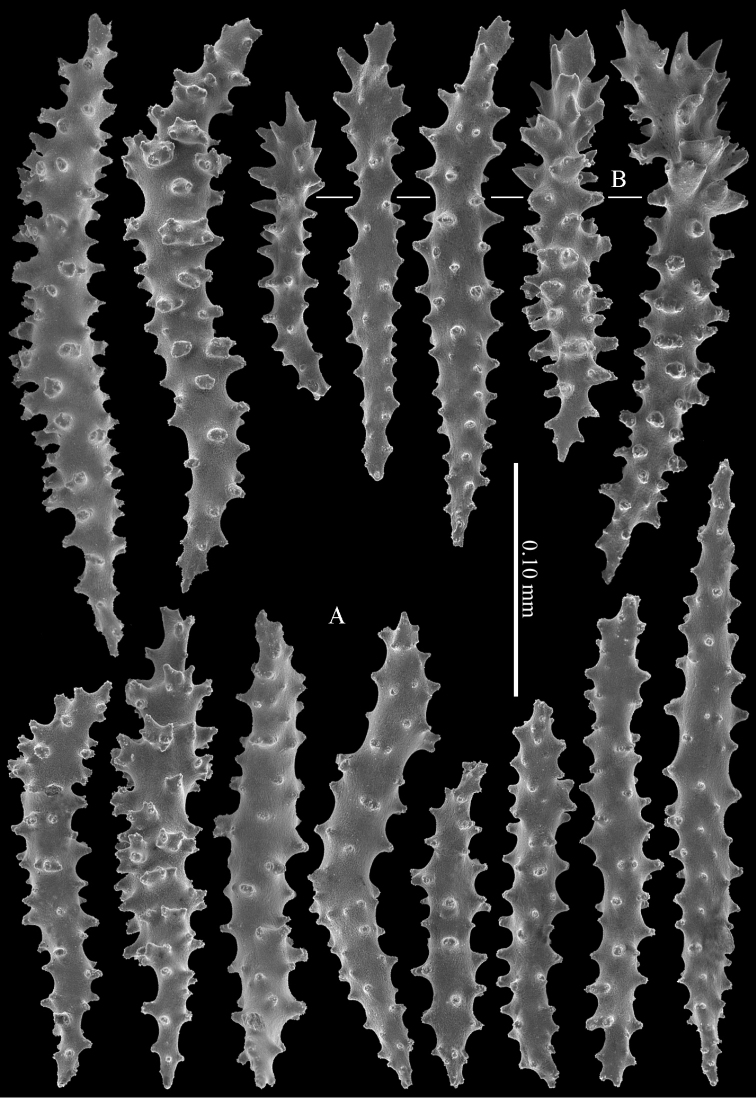
Coenenchymal sclerites of *Melithaea davidi* sp. n., holotype, RMNH Coel. 42122; **A** spindles **B** clubs.

**Figure 5.**
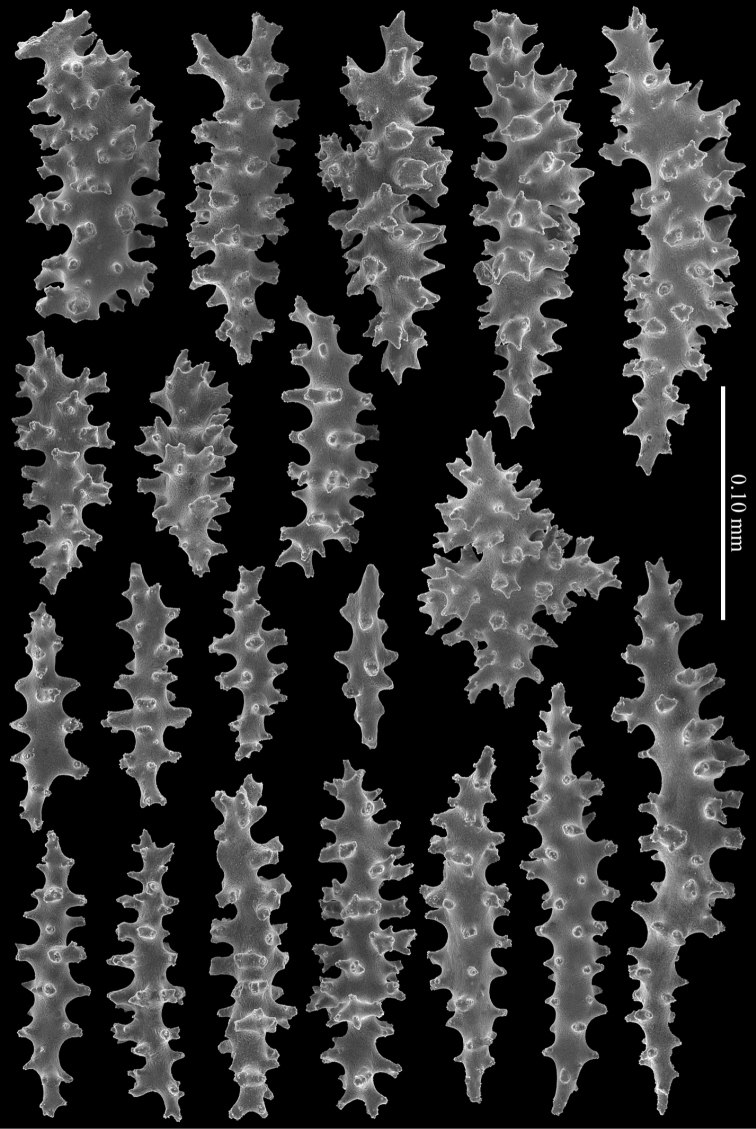
Coenenchymal sclerites of *Melithaea davidi* sp. n., holotype, RMNH Coel. 42122.

**Figure 6.**
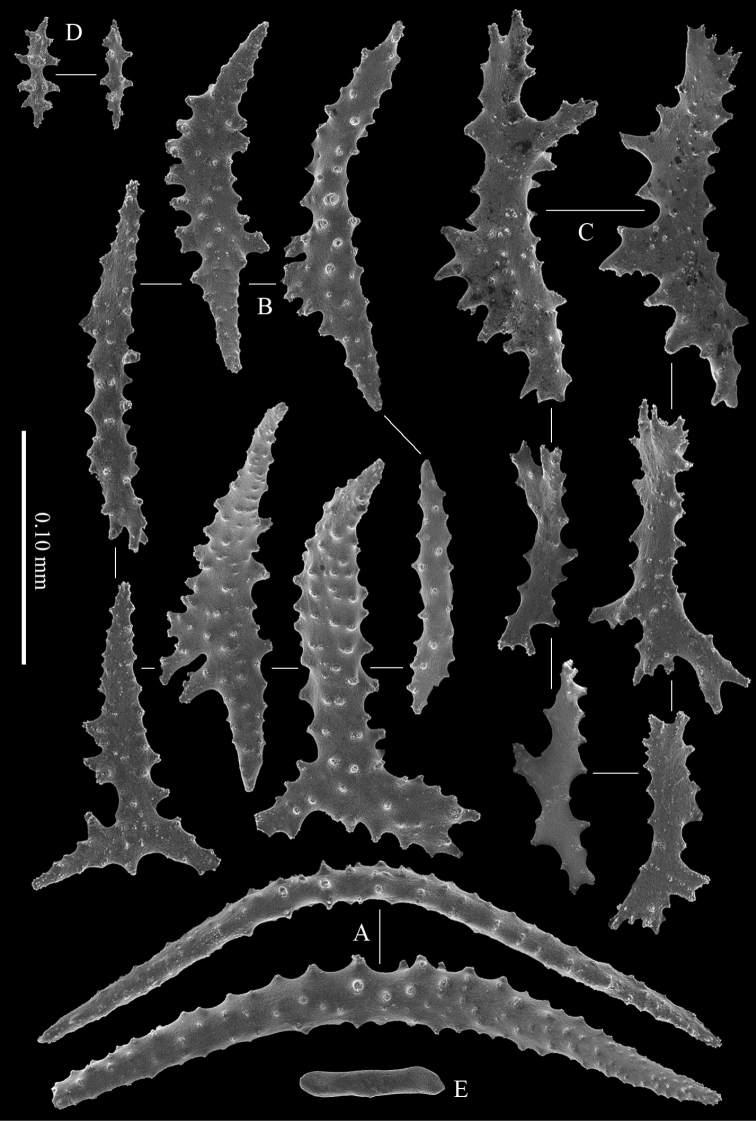
Polyp sclerites of *Melithaea davidi* sp. n., holotype, RMNH Coel. 42122; **A** collaret sclerites **B** point sclerites **C** tentacle sclerites **D** Pharynx sclerites **E** node sclerite.

**Figure 7.**
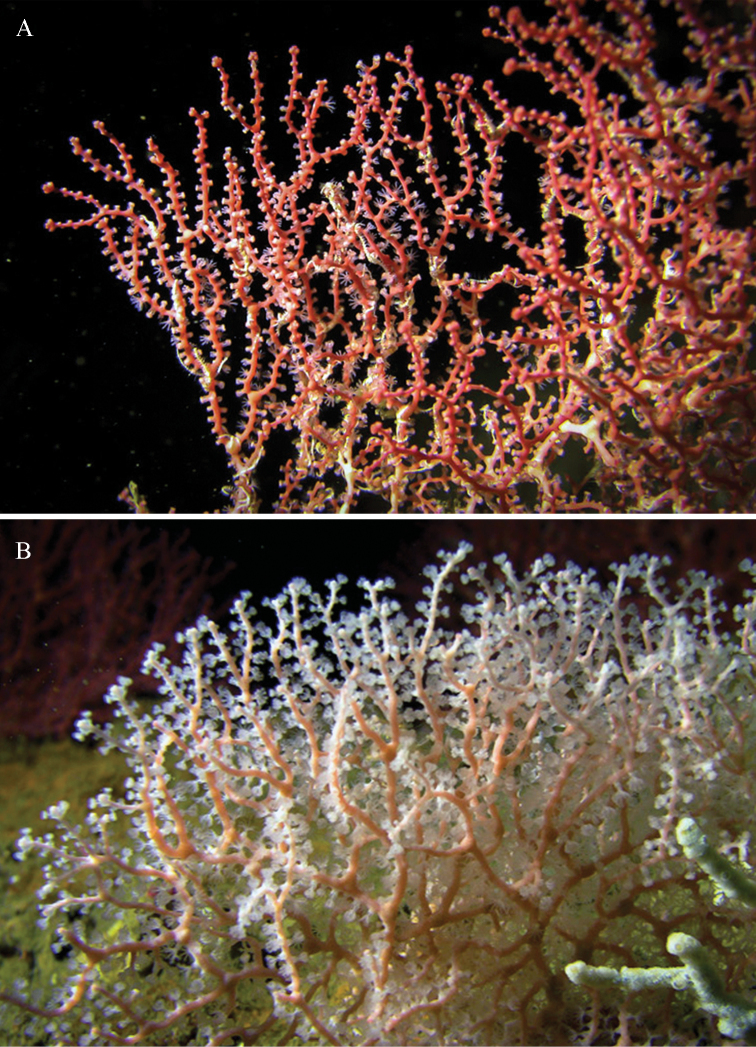
Underwater photographs of *Melithaea davidi* sp. n. at 79 m depth. **A** Holotype, RMNH Coel. 42122 **B** paratype, RMNH Coel. 42124.

Figures 3 , 4 , 5 , 6 , 7

##### Material examined.

*Holotype*: RMNH Coel. 42122, Oman, Oman Sea, 23.654267°N 58.629567°E, 79 m deep on a ship wreck, Robert’s barge, coll. David Mothershaw and Robin Norman, 19 July 2013. *Paratypes*: RMNH Coel. 42123, RMNH Coel. 42124, same data as holotype.

##### Description.

The holotype is branching dichotomously in several parallel planes, forming a network with many anastomoses. It is 12 cm high and 9 cm wide (Figure 3). The nodes are larger and more swollen in the basal parts of the colony. Many branches are covered with tiny white ophiuroids.

Polyp mounds and calyces are up to 1 mm in diameter. Calyces are projecting above the coenenchyme and are mostly situated along the sides of the branches. Polyps are situated 1–1.5 mm apart from each other (Figure 3).The coenenchyme has spindles, up to 0.35 mm long, with irregular simple tuberculation (Figure 4A). Additionally, the calyces have clubs, also up to 0.35 mm long, with simple tubercles and leaf-like projections at the distal end (Figure 4B). The calyx sclerites are mostly arranged en chevron. Some of the coenenchymal spindles have leaf-like or spinose side projections (Figure 5). In addition, there are some irregularly shaped sclerites present, up to 0.15 mm long, with or without leaf-like and spinose projections. Capstans are not present in the coenenchyme.

Polyps have two rows of collaret spindles and four spindles per point. The collaret spindles are up to 0.40 mm long, with more tuberculation on the middle of the convex side, and less tuberculation at the distal ends (Figure 6A).

The point sclerites are up to 0.25 mm long, with simple tubercles and projecting spines at the distal end (Figure 6B).

The tentacles contain flattened, dragon-wing shaped sclerites up to 0.15 mm long (Figure 6C).

The pharynx and introvert have small spiny sclerites that are up to 0.05 mm long (Figure 6D).

The nodes and internodes have internal rods and cigar-shaped sclerites up to 0.12 mm long, with or without median whorl of projections (Figure 6E).

##### Etymology.

The species is named after David Mothershaw who collected the specimens.

##### Colour.

The holotype is orange-red (Figure 3A). The colour of the nodes in younger parts of the colony is the same as the colony colour but in the older basal parts of the colony, they are brownish. All sclerites are reddish. The live colony had the same colour as the preserved one, with whitish translucent polyps (Figure 7).

##### Morphological variation.

One paratype (RMNH Coel. 42124) is light pink (Figures 3C, 7B).

##### Remarks.

The species resembles *Melithaea biserialis* (Kükenthal, 1908) and *Melithaea sinaica* Grasshoff, 2000, both described from the nearby Red Sea. *Melithaea biserialis* and *Melithaea sinaica* both have more tuberculate sclerites and, additionally, capstans that are not present at all in *Melithaea davidi*. The species also resembles *Acabaria* spec. indet. 2 Ofwegen (1987) from West India. However, that species also has capstans that are absent in *Melithaea davidi*.

*Acabaria* indet. 2 Ofwegen (1987) might represent a new species, however, the material is not sufficient for describing a new species.

*Acabaria mabahissi* Hickson, 1940, off Cape Guardafui, Gulf of Aden, and the Arabian Sea is the same as *Acabaria* spec. indet. 1 (Ofwegen 1987) from Somalia and Kenya.

##### Distribution.

Known only from the type locality.

## Discussion

Reijnen et al. (2014) observed that melithaeid species appear to be grouped phylogenetically by geographical region, suggesting high regional endemicity in this family. Our re-analysis of their mtMutS and 28S sequence data reflects this pattern, with species from the western Indo-Pacific (Indonesia, Malaysia, Japan, Palau, etc.), east and south African coasts (Tanzania, South Africa), northern and western Indian Oceans (Seychelles, Maldives), and the Red Sea separated into distinct well-supported clades (Figure 2). Therefore, the likelihood of species having wide geographical ranges is low, and consequently we did not compare the new species with similar-looking species occurring in other geographical regions. The molecular phylogenetic analysis suggests that *Melithaea davidi* is closely related to but distinct from several other species found in the Red Sea region for which we had sequence data for comparison. Although we did not have sequence data for *Melithaea biserialis* or *Acabaria* spec. indet. 2 reported by Ofwegen (1987), morphological differences support the distinction of those species from *Melithaea davidi*.

## Supplementary Material

XML Treatment for Melithaea

XML Treatment for Melithaea davidi
